# Supplementary material for: Chromothripsis during telomere crisis is independent of NHEJ, and consistent with a replicative origin
Source: Genome Res. 2019 May;29(5):737–49. doi: 10.1101/gr.240705.118 (PMC6499312; doi:10.1101/gr.240705.118)
Supplement: Supplemental Material [file supp_gr.240705.118_Supplemental_file_1.zip › contigs/annotated_contigs/DB78/contig.2.DB78_length_391_mean_cov_8.82352941176.docx]

**DB78_length_391_mean_cov_8.82352941176**

```
TAGCTATCAAGTAATTTTGCAGAAACCTTCTGCCACACTCCTGTGGAGTGACCAAGGTTGGGTTTAATATGATGGAGCACTGTCTTTGA
 >chr22:48722376-48722514 + E=1e-65
GCAGCGAGATTGGATTTGGCATCATTGCCATGTCTGTCAGAGATTTTG|A|CAGTTCAGCTGCCCAAGAGGAGCTCAATCCTGCCAGCA
                                                 >chr22:48725572-48725826 + E=1e-141
GCAACATGTGAGCCTGACAGCACATTATTTCCCGCTCAAGCTTTCAGATGAAACAGCAGCCACAATAGGAGGCTTAGCTGCGACTTCAT

CAGGGATCACAAGCTGGAGGATTTAGCCAAGTGCACATGAATTTCTGACTCACAGAGACTGAGATAACAAGGGCTTATTGTTTAAACTG

CTAAAGTGTGAGATGACGTGTTATTAGGCTACACATA
```
